# Isolation‐by‐distance and isolation‐by‐oceanography in Maroon Anemonefish (*Amphiprion biaculeatus*)

**DOI:** 10.1111/eva.13448

**Published:** 2022-08-25

**Authors:** Kyra S. Fitz, Humberto R. Montes, Diane M. Thompson, Malin L. Pinsky

**Affiliations:** ^1^ Department of Ecology, Evolution and Natural Resources Rutgers University New Brunswick New Jersey USA; ^2^ Institute of Tropical Ecology and Environmental Management Visayas State University Baybay City Philippines; ^3^ Department of Geosciences University of Arizona Tucson Arizona USA

**Keywords:** clownfish, connectivity, dispersal, marine conservation, marine larvae, population genetics

## Abstract

Obtaining dispersal estimates for a species is key to understanding local adaptation and population dynamics and to implementing conservation actions. Genetic isolation‐by‐distance (IBD) patterns can be used for estimating dispersal, and these patterns are especially useful for marine species in which few other methods are available. In this study, we genotyped coral reef fish (*Amphiprion biaculeatus*) at 16 microsatellite loci across eight sites across 210 km in the central Philippines to generate fine‐scale estimates of dispersal. All sites except for one followed IBD patterns. Using IBD theory, we estimated a larval dispersal kernel spread of 8.9 km (95% confidence interval of 2.3–18.4 km). Genetic distance to the remaining site correlated strongly with the inverse probability of larval dispersal from an oceanographic model. Ocean currents were a better explanation for genetic distance at large spatial extents (sites greater than 150 km apart), while geographic distance remained the best explanation for spatial extents less than 150 km. Our study demonstrates the utility of combining IBD patterns with oceanographic simulations to understand connectivity in marine environments and to guide marine conservation strategies.

## 1 INTRODUCTION

Many marine organisms produce offspring that have the potential to disperse long distances. This phenomenon of larval dispersal is difficult to measure and understand because direct observation or mark‐recapture tagging of marine larvae is costly and time‐intensive or even impossible. Understanding and accurately measuring marine larval dispersal is of considerable interest, however, as larval dispersal plays a substantial role in population dynamics and persistence over time (Hastings & Botsford, 2006; McManus et al., 2020), community assembly and dynamics (Gaines & Roughgarden, 1985; Leibold et al., 2004), local adaptation (Bradbury et al., 2008; Kawecki & Ebert, 2004; Kleypas et al., 2016; Lenormand, 2002; Walter et al., 2009), range expansion and recovery following disturbance (Magris et al., 2014; Treml & Halpin, 2012; Wood et al., 2016), and ecosystem function and resilience (Nyström & Folke, 2001). Accurate estimates of larval dispersal are also crucial to developing effective marine conservation strategies and successfully managing fisheries (Jones et al., 2007; Kough et al., 2013; Saura et al., 2014).

Dispersal in the ocean can be represented by a dispersal kernel in which the height of the kernel indicates the probability density of dispersal to a particular location relative to the parents. Some larvae will stay close to their natal location, while others disperse farther away (Nathan et al., 2012). The challenge facing researchers today is understanding the width and shape of the dispersal kernel. Examples of long‐distance marine larval dispersal (hundreds to thousands of km; Mora & Sale, 2002; Wood et al., 2016) and short‐distance dispersal (a few km; Almany et al., 2017; D'Aloia et al., 2013; Taylor & Hellberg, 2003) demonstrate the wide range in dispersal among marine species and among ocean regions. Further, theory predicts that dispersal kernels will differ among species that inhabit fragmented versus continuous habitats (Baskett et al., 2007; Shaw et al., 2019). Species in fragmented or patchy habitats are predicted to evolve shorter dispersal than species in continuous habitats, but this theory remains poorly tested in the field (Baskett et al., 2007; Shaw et al., 2019).

Multiple genetic methods have been developed to evaluate larval dispersal using neutral molecular markers. An increasingly popular genetic estimate for larval dispersal uses isolation‐by‐distance (IBD) patterns (Rousset, 1997). IBD refers to the increase in genetic differentiation among populations as the geographic distance among them increases. The slope of this relationship reflects the width of the larval dispersal kernel, with steeper IBD slopes in species with narrower dispersal kernels (Rousset, 1997; Wright, 1943). These IBD patterns result from the balance between genetic drift and migration, and steeper slopes reflect either low migration or low effective population density. Observed IBD patterns largely represent dispersal over the last few generations when sampling is done over spatial extents on the order of 10 to 50 times the dispersal spread (Hardy & Vekemans, 1999; Palumbi, 2003; Slatkin, 1993). This is a relevant timescale for ecological analyses and for informing conservation actions based on present conditions. As at least half of the marine organisms studied to date show IBD patterns, these patterns provide a method for estimating larval dispersal that can be widely applied across taxa (Selkoe et al., 2016; Wright et al., 2015). Efforts to validate IBD estimates against direct dispersal observations in anemonefish and neon gobies suggest congruent answers (Naaykens & D'Aloia, 2022; Pinsky et al., 2017), but congruence remains to be investigated in most marine species.

Ocean currents are a primary driver of marine larval dispersal, and some studies have reported that patterns of ocean currents explain the genetic differentiation among populations substantially more effectively than geographic distance (Schunter et al., 2011; Watson et al., 2011; White et al., 2010). This may depend on the spatio‐temporal heterogeneity of ocean currents. If advection and diffusion by ocean currents is consistent across space, larval transport may be fairly uniform and IBD theory will likely hold true (Cowen et al., 2006). Conversely, in areas with substantial differences in ocean current speed or direction among locations, patterns of dispersal may not be uniform among populations (Cowen et al., 2006). Comparing geographic distance and oceanographic connectivity allows us to evaluate the extent to which IBD patterns are apparent, which may vary between species or within species based on ocean currents and island geography (Bradbury & Bentzen, 2007; Selkoe et al., 2016). These effects may also be scale‐dependent, with more uniform dispersal at finer scales and ocean current impacts more apparent at wider scales (Benestan et al., 2021; Saenz‐Agudelo et al., 2015).

Our study investigates dispersal in an Indo‐West Pacific reef fish, *Amphiprion biaculeatus* (Maroon Anemonefish). *A. biaculeatus* is a relatively rare reef fish in the region, contrasting with previous dispersal studies that have focused on more common species such as the anemonefish *Amphiprion clarkii* and *Amphiprion percula* (Abesamis et al., 2017; Almany et al., 2007; Harrison et al., 2012; Pinsky et al., 2010; Saenz‐Agudelo et al., 2012). It also has more specific habitat preferences (*A. biaculeatus* only settles in monogamous pairs on unoccupied *Entacmaea quadricolor* anemones) than the more common anemonefish *A. clarkii* and *A. percula*, both of which will settle on multiple species of anemone and can have more than two fish per anemone. *A. biaculeatus'*s highly specific habitat preferences resemble a patchy or fragmented habitat, while *A. clarkii* and *A. percula*'s generalist habitat preferences resemble more continuous habitats. The differences in habitat preferences among species make anemonefish a useful study system for testing the theoretical prediction of species evolving shorter dispersal in fragmented habitats.

In this study, we address the questions: (1) What is the dispersal spread of *A. biaculeatus*, and does it follow theoretical predictions of being shorter than the dispersal spread of species in less fragmented habitats? and (2) Are IBD patterns or isolation‐by‐oceanography patterns more relevant for *A. biaculeatus* dispersal, and does this depend on spatial extent? For question 1, estimates of effective population density were generated and applied to IBD theory to calculate estimates of dispersal. We expected to find shorter dispersal spread in *A. biaculeatus* than other species of anemonefish due to *A. biaculeatus* having specialized habitat preferences that increase habitat patchiness and fragmentation in comparison with the greater habitat options for other anemonefish. For question 2, we tested whether estimates of potential connectivity based on ocean current velocity were better predictors of genetic differentiation in *A. biaculeatus* at two spatial extents. We predicted IBD patterns would be more relevant for dispersal over narrow regions with homogenous ocean current patterns, while isolation‐by‐oceanography patterns would be apparent over wider spatial extents with heterogeneous ocean current patterns.

## 2 MATERIALS AND METHODS

### 2.1 Study system

*A. biaculeatus* (Maroon Anemonefish) is a species distributed from the Indo‐Malaysian archipelago to northern Queensland, Australia (between 28°N–26°S and 83°E–178°W). *A. biaculeatus* was previously classified as *Premnas biaculeatus*, and evidence from molecular phylogenies has shown the validity of classifying this genus together with the remaining anemonefish in the *Amphiprion* genus (Frédérich et al., 2013; Litsios & Salamin, 2014; Tang et al., 2021). They are protandrous hermaphrodites and are monogamous, with two adults per anemone (Mariscal et al., 1993). *A. biaculeatus* lives exclusively with the anemone *Entacmaea quadricolor*. Eggs are laid by the female next to the parent's anemone and take 7 days to hatch (Mariscal et al., 1993). Larvae spend 7–11 days in the ocean before settling onto an anemone (Mariscal et al., 1993). Anemonefish are a group of conservation interest due to their exploitation for the aquarium trade, and *A. biaculeatus* has been identified as a species especially vulnerable to overcollection, making it an ideal candidate for spatial management such as the implementation of marine reserves (Dee et al., 2019).

**FIGURE 1:**
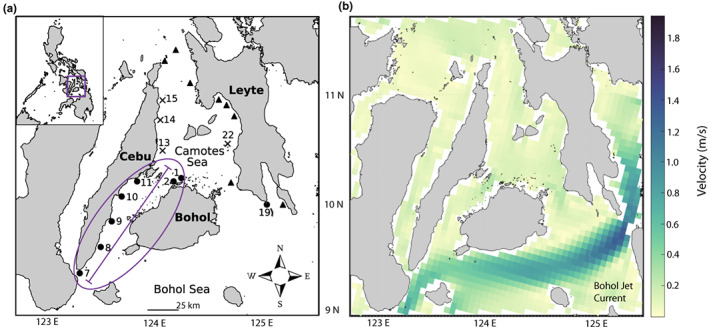
(a) Map of Cebu, Bohol, and Leyte showing the location for each sampling site. Sites 13, 14, 15, and 22 (shown with an “X”) were excluded from further analysis due to sample sizes of less than five individuals. The triangle symbol denotes sites that were surveyed but at which no *A. biaculeatus* were found. The purple oval is referred to as our IBD study region, and the purple line represents the length of the IBD study region. (b) Plots of mean velocity from January 2003 to December 2007 in m/s. The strongest currents in our study region flow in the Bohol Sea from northeast to southwest and are known as the Bohol Jet Current.

Our study focused on *A. biaculeatus* populations inhabiting coral reefs along the islands of Cebu, Bohol, and Leyte in the central Philippines. The region has fairly continuous coral reefs, with some interruptions near river outflows and patchier reefs on Leyte. Oceanographic measurements and modeling suggest that currents in the region are strongest to the south of Bohol where the Bohol Jet Current flows east to west in the Bohol Sea (Figure 1; Gordon et al., 2011). The jet is driven by the inflow of water from the western Pacific. Currents are slowest in the shallow straits between Cebu and Bohol (Figure 1).

Study sites 25 km apart were selected along the east coast of Cebu (*n* = 8), the north coast of Bohol (*n* = 2), and the west coast of Leyte (*n* = 2; Figure 1). We used spatial extents only up to 210 km to investigate recent migration and fine‐scale dispersal, as genetic patterns over smaller spatial extents are more likely to represent dispersal over the past few generations and less likely to be influenced by deeper time evolutionary events (Hardy & Vekemans, 1999; Rousset, 1997; Slatkin, 1993).

### 2.2 Visual surveys

Two scuba divers measured census density of *A. biaculeatus* between August and October 2008 by swimming underwater visual transects parallel to the reef at depths between 3 and 12 m. They recorded the number and size of anemonefish on each anemone found in two 5‐m swaths. We chose survey locations 25 km apart by GPS prior to visiting the site so that we estimated an unbiased, mean coastal density (*D*). We did not relocate transects if habitat was sand, mud, or otherwise poor. A towed GPS unit on the surface recorded diver position every 15 s in order to measure transect lengths. Transects averaged 655 ± 51 m long.

### 2.3 Genetic sampling

We captured fishes with drive and dip nets while on SCUBA at the sampling sites from August–October 2008 and collected nonlethal fin clips underwater from the first 20 fish per site. If fish were not found at the visual survey location, we searched nearby locations with higher habitat quality (e.g., a nearby coral reef) within the same Local Government Unit for up to 2 days. Samples were stored in 70% ethanol, and GPS location for each sample was recorded.

DNA was extracted from all samples with Nucleospin (Machery‐Nagel) or DNEasy 96 (Qiagen) column extraction kits. Sixteen microsatellite loci were amplified and genotyped (Table 1). We found eight loci through cross‐species amplification of loci screened by Beldade et al. (2009) but not published by them (G. Bernardi, pers. comm., see Table S1 for the primer sequences). The 10 μl PCR reactions contained 1 μl genomic DNA, 1× Fermentas PCR buffer, 3 mM MgCl_2_, 500 nM fluorescently labeled primer, 500 nM unlabeled primer, 40 μM each dNTP, and 0.1 μl (0.5 U) Fermentas Taq. We did not quantify DNA concentration before beginning PCR. Thermal cycling consisted of a 94°C denaturing step for 2 min, followed by 30 cycles of 94°C for 45 s, annealing temperature for 45 s (Table 1), and 72°C for 45 s, followed by a final extension at 72°C for 1 min. PCR products were genotyped on an Applied Biosystems 3730 (MRDDRC Molecular Genetics Core Facility at Children's Hospital Boston) and analyzed in GeneMapper 4.0 (Applied Biosystems).

**TABLE 1:** Microsatellite loci used in this study

Locus	Annealing temperature (°C)	No. of alleles	*H*_e_	*p* (*p*‐value for departure from HWP)	References
AC1359	53	4	0.481	0.33	Liu et al. (2007)
AC1578	53	4	0.501	0.35	Liu et al. (2007)
APR_Cf29	53	11	0.721	0.29	Buston et al. (2007)
NNG_012	53	4	0.453	0.32	Watts et al. (2004)
ACH_A11	53	17	0.815	0.92	G. Bernardi pers. comm.
ACH_A4	53	7	0.723	0.49	G. Bernardi pers. comm.
ACH_B9	53	11	0.777	0.06	G. Bernardi pers. comm.
NNG_028	53	19	0.652	0.38	Watts et al. (2004)
ACH_A7	53	5	0.450	0.28	G. Bernardi pers. comm.
ACH_C1	53	7	0.660	0.8	G. Bernardi pers. comm.
ACH_D1	53	19	0.867	0.87	G. Bernardi pers. comm.
ACH_A3	58	2	0.454	0.95	G. Bernardi pers. comm.
ACH_A8	57	5	0.658	0.33	G. Bernardi pers. comm.
NNG_004	53	4	0.224	0.83	Watts et al. (2004)
NNG_007	58	6	0.619	0.15	Watts et al. (2004)
APR_Cf39	60	7	0.715	0.98	Buston et al. (2007)

### 2.4 Genetic analysis

Sites without any *A. biaculeatus* were excluded from further analysis, as were four sites (13, 14, 15, and 22) with sample sizes less than five individuals (Figure 1). We conducted statistical analysis in R Studio version 1.4.1106 using R version 4.0.5 (R Core Team, 2021). We used a significance threshold of *p* < 0.05 to reject the null hypothesis in all of our statistical tests. We considered a *p*‐value between 0.05 and 0.1 to be marginally significant. We assessed departure from Hardy–Weinberg proportions (HWP) independently for each locus within each site and calculated pairwise *F*_ST_ between sites using the Genepop package version 1.1.7 (Raymond & Rousset, 1995; Rousset, 2008).

We evaluated the presence of an IBD pattern by running a Mantel test on matrices of pairwise geographic distance and pairwise linearized *F*_ST_ (i.e., *F*_ST_/[1 − *F*_ST_]). Pairwise geographic distance was measured using the ruler tool in Google Earth as the shortest distance between sites that did not cross land. We used the vegan package version 2.5‐7 (Oksanen et al., 2020) to run the Mantel test and an ordinary least squares linear regression in R to calculate the slope of the relationship between geographic and genetic distance. Mantel tests were set to 999 permutations, unless the package automatically corrected to a different number of minimum permutations.

### 2.5 Effective density

We used the linkage disequilibrium method in the program NeEstimator (Do et al., 2014) to estimate the effective number of breeders (*N*_b_) with a jackknife confidence interval. We only included sites in the region found to follow an IBD pattern (IBD study region in Figure 1). We selected a monogamous mating model and a critical allele frequency (*P*_crit_) of 0.02, following Waples and Do's (2010) rule of thumb of *P*_crit_ = 0.02 for sample sizes greater than 25.

To estimate *N*_e_, we calculated the *N*_b_/*N*_e_ ratio to account for the presence of overlapping generations using Waples et al.’s (2014) equations. These equations take into consideration the expected lifespan and the minimum age of reproduction. We used the FishLife package (Thorson et al., 2017) to estimate a lifespan of 7.05 years and a minimum age of reproduction of 1.68 years. Finally, we divided the *N*_e_ estimate by the length of the IBD study region measured in ArcGIS (130 km) to approximate the linear effective density estimate (*D*_e_).

For comparison against *D*_e_, we also calculated census density. We measured the area and length of our IBD study region (Figure 1) using the World Ocean Base layer in ArcGIS online (ESRI 2014). We included areas with depth up to 12 m, as this was the maximum depth reached in the visual surveys. To calculate the linear census density (fish/km) in the IBD study region, we took the average density of *A. biaculeatus* individuals observed on the visual surveys (fish/km^2^), multiplied it by the area of the reef in the study region (648 km^2^), and divided by the length of the study region (130 km). We counted both individuals on each anemone in this census density because both fish on each anemone are breeding adults in *A. biaculeatus*. We calculated 95% confidence intervals by bootstrapping with 1000 replicates across visual surveys and using the adjusted bootstrap percentile (BCa) method.

### 2.6 IBD

In a continuous population, the balance between genetic drift and migration leads to a positive correlation between pairwise genetic and geographic distances among individuals or populations. This correlation is called IBD, and it can be used to estimate dispersal distance (Rousset, 1997). Specifically, one can estimate dispersal spread (σ), which is the standard deviation of the dispersal kernel. The dispersal kernel is a probability distribution where height is the probability density of a larva dispersing to a certain position relative to its parents. The spread is also the same as the standard deviation of parental position relative to the offspring position (Siegel et al., 2003). Rousset (1997) demonstrates that in a 1D habitat (where populations are separated by a distance longer than the width of the habitat), the dispersal kernel spread (σ) can be calculated as

$$\sigma =\sqrt{\frac{1}{4D_{e}m},}$$

where *D*_e_ is the linear effective population density and *m* is the slope of the regression between linearized *F*_ST_ and geographic distance. Our study region and the coral reefs that *A. biaculeatus* inhabit are longer than they are wide (length is greater than 100 km, while width is less than 10 km), and the distance between populations is greater than the width of the habitat (25 km between sampling sites, while width is less than 10 km) and so approximate a 1D habitat. We propagated error for our estimate of σ by sampling 1 million times from the uncertainty in each parameter. We used a normal distribution fit to the mean and standard error of the *N*_b_ */N*_e_ ratio (mean = 0.958; SD = 0.186) and used a chi‐squared error distribution fit to the lower 95% confidence interval for *N*_b_ (we did not obtain a finite upper bound for *N*_b_). We represented *m* as a normal distribution fit to the mean and standard error of the IBD slope.

Calculating dispersal spread from IBD theory relies on a few assumptions. First, effective population density (*D*_e_) should be roughly consistent across sites. Second, dispersal spread and patterns should have been stable for multiple generations, and the populations should be at or near drift‐migration equilibrium (Rousset, 1997). IBD theory also assumes no selection, a negligible mutation rate, and is based on a Wright‐Fisher model of reproduction (Wright, 1931). The Wright–Fisher model assumes constant population size, nonoverlapping generations, random mating, an equal sex ratio, and no selection or mutation (S. Wright, 1931). Our study system meets these assumptions reasonably well. For our IBD analysis, we excluded sites to the north where *A. biaculeatus* was difficult to find and likely has a lower effective population density. We sampled across a spatial extent likely 10σ–50σ, and so the populations should be at or near drift‐migration equilibrium (Hardy & Vekemans, 1999; Vekemans & Hardy, 2004). Microsatellites are not typically under selection, though we also evaluated HWP. The monogamy of breeding pairs is of short‐enough duration to have limited impact on random mating assumptions. The sex ratio is fairly equal in *A. biaculeatus*, and there is no evidence for assortative mating. We do likely have overlapping generations, violating the assumption of nonoverlapping generations. We addressed this by applying Waples et al.’s (2014) correction equations when generating our estimate of effective density. The difference between our initial and corrected effective density estimates was small.

### 2.7 Potential connectivity analyses

We used potential connectivity matrices from Thompson et al. (2018) to assess the probability of larvae dispersing between pairwise reef sites based on ocean currents. Potential connectivity refers to the physical drivers that influence larval transport, such as current velocity (and variability), and is calculated as the probability of larval transport at the end of the pelagic larval duration (PLD) from a source to a sink reef (Watson et al., 2010). Connectivity matrices were generated by running the (offline) Lagrangian particle tracking tool TRACMASS (Döös et al., 2013) with the zonal and meridional currents simulated on a 5 km by 5 km grid in a Regional Ocean Modeling System developed for the Coral Triangle region (Castruccio et al., 2013). TRACMASS parameters were specified for a model organism with a 10‐day PLD. The connectivity matrix we used was an average of historical simulations from 1960 to 2006 using particle releases at midnight for 5 days surrounding the full moons in April and September of each year. These parameters are a reasonable but not perfect approximation for *A. biaculeatus*, which has a PLD of 7–11 days and mates year‐round (Mariscal et al., 1993).

Twenty‐five particles were released from each 5 × 5 km reef or coastal grid cell (i.e., those located within two grid cells of the coastline or coral reef) for each day of the 5‐day spawning event and were tracked to and from 40 × 40 km release sites (i.e., 8 × 8 coastal cells, grouped to reduce computational cost). Thus, a total of around 8000 particles were tracked for each release site, with the exact number varying with the proportion of oceanic and land cells within the release site. The grid size is five times smaller than the distance between our sampling sites. Sensitivity analyses of particle numbers (between 1 and 50,000) demonstrate that 8000 particles captured ~95% of the variance in the Lagrangian probability density function, calculated as the particle density at the end of the PLD (normalized by the number of particles from each release site). This approach was, therefore, sufficient to capture the potential connectivity patterns in this region while optimizing the computational and storage demands of the simulations and analyses. Further, Thompson et al. (2018) demonstrate that connectivity patterns and source/sink strength are similar among neighboring release sites, suggesting that this resolution is sufficient to capture the spatial gradients observed across this region (see Thompson et al., 2018 for a complete description of connectivity simulations).

To evaluate the relationship between potential connectivity and genetic distance, we assigned each *A. biaculeatus* sampling site to the nearest particle release site. We then extracted potential connectivity values between each pair of sampling sites. The close proximity of sampling sites 1 and 2 (10 km apart) meant that one release site was chosen for both sampling sites. We, therefore, combined samples from sites 1 and 2 and calculated pairwise *F*_ST_ between this combined site and each of the rest of the sites.

We ran Mantel tests between pairwise potential connectivity and pairwise linear *F*_ST_ using the R package vegan to assess the correlation between passive dispersal via ocean currents and genetic distance. We also ran two partial Mantel tests: The first between pairwise potential connectivity and pairwise linear *F*_ST_ while controlling for geographic distance, and the second in the IBD region between pairwise geographic distance and pairwise linear *F*_ST_ while controlling for potential connectivity. Because site 19 was later found to be an outlier from the IBD pattern, we ran a linear regression between pairwise potential connectivity and pairwise linear *F*_ST_ for site 19 compared with each other site.

## 3 RESULTS

### 3.1 Genetic sampling and analysis

**FIGURE 2:**
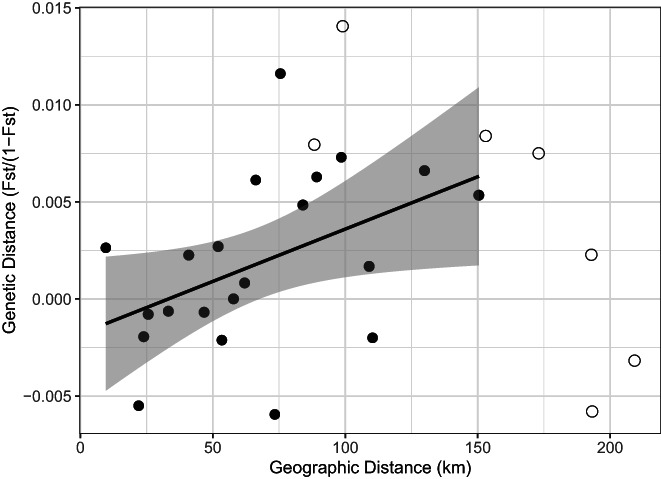
Plot of pairwise geographic distance and pairwise linearized genetic distance (*F*_ST_/[1 − *F*_ST_]) between sites. Pairwise comparisons involving site 19 (southern Leyte) are shown as unfilled circles and other comparisons as black dots. The line shows a linear regression without site 19, and the shaded region represents the 95% confidence interval around the slope.

*A. biaculeatus* individuals were relatively rare in our study region, particularly to the north where many sampling sites had no individuals (Figure 1; Table 2). Their host anemone, *E. quadricolor*, was found at all surveyed sites but, particularly to the north, was often occupied by other species of anemonefishes. In total, we genotyped 159 *A. biaculeatus* samples at 16 microsatellite loci (Table 2). However, four sites (13, 14, 15, and 22) were excluded from further analysis due to sample sizes less than five individuals (Figure 1; Table 2), leaving us with a sample size of 150. There were no significant departures at any locus from HWP, though ACH_B9 approached significance (Table 1). Pairwise *F*_ST_ between populations ranged from −0.006 to 0.014 (Figure 2).

**TABLE 2:** Sample size at each site with *A. biaculeatus*

Site number	Site name	Island	No. of *A. biaculeatus*
1	Getafe 1	Bohol	20
2	Getafe 2	Bohol	21
7	Santander	Cebu	16
8	Boljoon	Cebu	16
9	Argao	Cebu	17
10	Carcar	Cebu	21
11	Minglanilla	Cebu	19
13	Danao	Cebu	4
14	Sogod	Cebu	2
15	Tabogon	Cebu	1
19	Padre Burgos	Leyte	20
22	Inopacan	Leyte	2
		Total	159
		Total after low sample size exclusion	150
		Total in IBD study region	130

*Note*: Sites 13, 14, 15, and 22 were excluded from further analysis due to having sample sizes less than five individuals. The isolation‐by‐distance (IBD) study region included sites 1, 2, 7, 8, 9, 10, and 11.

### 3.2 IBD

A Mantel test between linearized *F*_ST_ and geographic distance for all remaining sites was not significant (*r* = 0.147; 95% CI = −0.239 to 0.493; *n* = 8 sites; *p* = 0.261; 999 permutations; linear regression slope ± standard error = 1.36 × 10^−5^ ± 1.79 × 10^−5^). However, the clearest departures from an IBD trend all involved site 19, which was the most distant sampling site (Figures 1 and 2). A Mantel test between linearized *F*_ST_ and geographic distance on all sites except 19 was significant with a weak positive correlation (*r* = 0.456; 95% CI = 0.031 to 0.742; *n* = 7 sites; *p* = 0.03; 5039 permutations; linear regression slope *m ± standard error* = 5.393 × 10^−5^ ± 1.663 × 10^−5^; Figure 2).

### 3.3 Effective density and dispersal distance

We calculated an effective number of breeders (*N*_b_) of 6942 individuals with a jackknife confidence interval of 779.5 to ∞ for the IBD study region (*n* = 130). Adjusting for overlapping generations, we calculated an *N*_e_ of 7625 individuals and a *D*_e_ of 58.7 fish/km (95% CI = 6.6 to ∞ fish/km). Our mean census density from visual surveys in the IBD study region, for comparison, was 1758 fish/km (95% CI = 997 to 2492 fish/km).

Using the IBD equation and propagating error through each parameter, we then calculated a point estimate of dispersal spread (σ) of 8.9 km and a 95% confidence interval of 2.3 to 18.4 km for the IBD study region. To compare our results to other anemonefish species, we calculated the fraction of samples from the *A. biaculeatus* dispersal spread (σ) uncertainty greater than or equal to dispersal spread estimates for *A. clarkii* (11 km; see Pinsky et al., 2010) and *A. percula* (17 km; see Pinsky et al., 2017). We found that 23% of *A. biaculeatus* values were greater than the 11 km dispersal spread of *A. clarkii*, while 3.8% were greater than the 17 km dispersal spread of *A. percula*.

### 3.4 Potential connectivity

**FIGURE 3:**
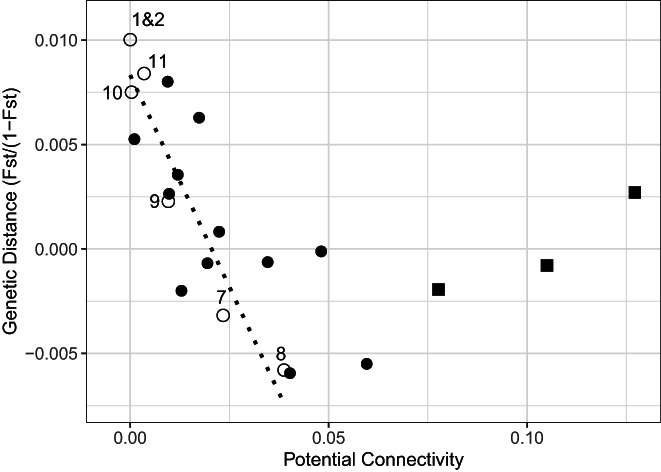
Plot of pairwise potential larval connectivity and pairwise linearized genetic distance (*F*_ST_/[1 − *F*_ST_]) between sites. Pairs of sites in the IBD study region are shown in black dots; pairs involving site 19 (southern Leyte) are shown in unfilled circles. Pairwise comparisons between sites 8&9, 8&10, and 9&10 (in southern Cebu) are shown as squares. The dotted line shows a linear regression for comparisons involving site 19 and each other site.

A Mantel test between pairwise potential connectivity and linear *F*_ST_ showed a moderately negative but nonsignificant correlation (*r* = −0.442; 95% CI = −0.734 to −0.012; *n* = 7 sites; *p* = 0.975; 5039 permutations; Figure 3). A partial Mantel test similarly revealed a moderately negative but nonsignificant correlation between potential connectivity and linear *F*_ST_ while controlling for geographic distance (*r* = −0.415; *n* = 7 sites; *p* = 0.963; 5039 permutations). A Mantel test run on sites in the IBD region (all sites except for 19) showed a moderately negative but nonsignificant correlation between potential connectivity and linear *F*_ST_ (*r* = −0.337; 95% CI = −0.724 to 0.212; *n* = 6 sites; *p* = 0.817; 719 permutations). A partial Mantel test in the IBD region and controlling for potential connectivity showed a moderately positive and marginally significant correlation between geographic distance and linear *F*_ST_ (*r* = 0.403; *n* = 6 sites; *p* = 0.099; 719 permutations). Most pairwise comparisons appeared to follow a negative linear relationship between potential connectivity and genetic distance, but, notably, all three pairwise comparisons of sites 8, 9, and 10 were outliers. These sites were all located in the shallow straits between Cebu and Bohol and in the middle of the IBD study region (i.e., where geographic distance explained genetic structure effectively). A Mantel test on all sites except 8, 9, and 10 revealed a strongly negative correlation but, with only four sites, the correlation was nonsignificant (*r* = −0.6616; 95% CI = −0.959 to 0.324; *n* = 4 sites, *p* = 0.917; 23 permutations).

Because we had found that site 19 did not follow an IBD pattern, we also assessed the correlation between potential connectivity and genetic distance between site 19 and each other site. We found a significantly strong, negative linear relationship between pairwise potential connectivity and pairwise linear *F*_ST_ from site 19 to the other sites (*R* ^2^ = 0.922; *p* = 0.0024; Figure 3).

## 4 DISCUSSION

Our research suggests that using IBD patterns and effective density can yield robust dispersal estimates at narrow spatial extents (less than 150 km), but that heterogeneous ocean currents can disrupt this pattern across wider spatial extents (greater than 150 km). We found IBD patterns in *A. biaculeatus* across reefs with relatively homogenous ocean currents, suggesting dispersal probabilities largely decline with geographic distance. In response to Question 1, we estimated a dispersal spread of around 9 km, significantly shorter than *A. percula* but similar to *A. clarkii* (Almany et al., 2007, 2017; Pinsky et al., 2010, 2017), at spatial extents less than 150 km. Our results appear to agree with our hypothesis of shorter dispersal spread in species with fragmented habitats. For Question 2, we found that ocean circulation explained observed genetic structure at wider spatial extents with more heterogeneous current speeds, while oceanographic models were less effective for explaining genetic structure in shallow ocean straits. This finding aligns with our hypothesis that IBD patterns would be more relevant at narrow spatial extents with homogenous current patterns and isolation‐by‐oceanography patterns would be relevant at wider spatial extents with heterogeneous current patterns. We show how IBD patterns may be used in tandem with ocean currents to better understand the drivers of marine larval dispersal.

### 4.1 Comparison with other coral reef species

Our 9 km dispersal spread estimate for *A. biaculeatus* is intermediate compared with other reef fishes and similar to other anemonefish species. Previous IBD dispersal spread estimates include an 11 km dispersal spread in *Amphiprion clarkii* from the same region of the Philippines (Pinsky et al., 2010) and 17 km dispersal spread in *Amphiprion percula* from Papua New Guinea (Pinsky et al., 2017). Genetic parentage methods have identified dispersal spreads that varied from 9 to 250 km across individual years for *A. clarkii*, but with an 11 km average spread, also in the central Visayas region of the Philippines (Catalano et al., 2021). A separate study on *A. percula* using genetic parentage methods in Papua New Guinea found the dispersal spread to be 27 km in 2009 and 19 km in 2011 (Almany et al., 2017). All of these estimates suggest that *A. biaculeatus* has a similar but lower dispersal spread than related species (particularly *A. percula*), perhaps because larval behavior favors shorter distance dispersal in order to remain near its relatively more rare host anemone. Our results, therefore, appear to agree with theoretical results predicting shorter dispersal in species with fragmented habitats (Baskett et al., 2007; Shaw et al., 2019). However, there may be additional processes occurring other than only *A. biaculeatus's* specialist preferences. For example, other species of anemonefish may be outcompeting *A. biaculeatus* during larval settlement in a process known as pre‐emptive competition, or competition for space as a limiting resource. For this process to explain the differences in dispersal spread, competition would have to be particularly detrimental for the survival of long‐distance dispersing *A. biaculeatus* larvae.

Because our estimate of dispersal spread comes from regions with moderately strong ocean currents (current velocity mean ± standard deviation = 0.258 ± 0.200 m/s), we recognize that dispersal spread could be lower (higher) in regions with weaker (stronger) currents. Further efforts comparing dispersal kernels across regions of different current speeds will be needed to test this conjecture. However, as estimates from other anemonefish taken in multiple locations are relatively similar, our estimate should be useful for conservation and management across the Indo‐Pacific.

In terms of other coral reef species, *A. biaculeatus* has intermediate dispersal distances. The squaretail coral grouper (*Plectropomus areolatus*) has a similar dispersal spread of 18 km (Almany et al., 2013). In contrast, the neon goby, *Elacatinus lori*, has a substantially shorter dispersal spread of 3.9 km (D'Aloia et al., 2015). Other coral reef species have much longer dispersal spreads than anemonefish, including the vagabond butterflyfish (*Chaetodon vagabundus*) at 42 km in the central Philippines (Abesamis et al., 2017) and 311 km in Papua New Guinea (Almany et al., 2017), the leopard coral trout (*Plectropomus leopardus*) at 225 km, and the bar‐cheek coral trout (*Plectropomus maculatus*) at 380 km (Williamson et al., 2016). While not directly comparable to kernel dispersal spread estimates, observations of the bicolor damselfish (*Stegastes partitus*) found a mean dispersal of 77 km (Hogan et al., 2012). Despite their unique association with anemones, anemonefishes do not appear to have unusual larval dispersal patterns.

We recognize that our methods have limitations. Microsatellites are useful for understanding population structure because of their high allelic diversity but represent only a small fraction of the genome. Genotyping thousands of single nucleotide polymorphisms or doing whole‐genome sequencing would provide the ability to detect selection acting on small fractions of the genome and provide greater precision in gene flow estimates, particularly in situations with weak genetic structure. The number of sites used in our IBD analysis (*n* = 7 sites) and in total (*n* = 8) may also be a limitation, particularly because our isolation‐by‐oceanography results relate in particular to one site. Our sampling results highlight *A. biaculeatus* as a rare species, with six sites yielding *A. clarkii* but no *A. biaculeatus* samples, and four sites yielding less than five *A. biaculeatus* individuals each. Limitations on the number of sites and sample sizes are a reality when studying rare species that are of interest for conservation, as the species of interest may have a low population size or only be found over a small area. We cannot infer whether IBD patterns exist outside our study area. As the 95% confidence interval for *N*_e_ is much wider than it is for the IBD slope, uncertainty in estimating *N*_e_ remains the greatest uncertainty when estimating dispersal spread. Thus, refining linkage disequilibrium methods for estimating *N*_e_ remains more important to generating accurate dispersal estimates than adding additional sampling sites. In our potential connectivity analyses, additional sampling sites would be useful for testing the role of ocean currents across a wider area with greater precision.

### 4.2 Effective and census density

Our estimates of effective density and census density in the IBD study region differed substantially, with an effective density estimate of 58.7 fish/km (95% CI = 6.6 to ∞ fish/km) and a census density estimate of 1758 fish/km (95% CI = 997 to 2492 fish/km). Finding a census density about an order of magnitude higher than effective density is common (Frankham, 1995; Waples et al., 2018), including for anemonefishes (Pinsky et al., 2010, 2017). In IBD populations, a lower effective density estimate is expected since gene flow among slightly diverged populations generally produces a Wahlund effect, increased linkage disequilibrium, and therefore a lower *N*_e_ estimate (Neel et al.,^2013^). A strong variance in reproductive success also contributes to *N*_e_ being lower than census population size, particularly in marine species (Hare et al., 2011; Waples et al., 2013). Further, our visual surveys may have been in areas where *A. biaculeatus* were more abundant than average, thus biasing our census densities higher despite efforts to choose survey sites without regard to habitat quality. In reality, *A. biaculeatus* densities are not uniform across all coastal areas, as we assumed when we measured the area of coastal habitat in our study region. In Bohol, where the area of the reef is higher than in Cebu, we only had one visual survey, which is likely not representative of all reefs off the coast of northwest Bohol.

Additionally, we were not able to obtain a finite upper bound to our estimate of *N*_e_. Precisely estimating *N*_e_ in populations with *N*_e_ > 1000 with genetic markers remains a challenge, even with larger sample sizes (Marandel et al., 2019; Waples & Do, 2010). While obtaining a finite upper bound for the 95% confidence interval was not possible with our data, we were confident in the lower bound, as linkage disequilibrium signals for large and small populations differ substantially (Waples & Do, 2010).

IBD assumes the effective density between sites is relatively consistent. It appears that we met this assumption because we only included sites in our analysis where *A. biaculeatus* individuals were relatively abundant and excluded sites to the north where they were hard to locate. However, dispersal spread can be underestimated when sampling is conducted in areas of high density that are surrounded by areas of low density (Leblois et al., 2004). Our dispersal spread estimate may, therefore, be a bit too low, though additional sampling to the south, east, and west would be needed to understand whether density is lower in all directions. Even if dispersal spread is underestimated, our conservation and management recommendations will be effective, as having a smaller than needed distance between reserves will still produce spillover effects.

### 4.3 Potential connectivity analyses

Marine fish disperse on ocean currents, and many studies find that ocean currents rather than geographic distance provide a more effective explanation for dispersal patterns (Schunter et al., 2011; Sefc et al., 2020; White et al., 2010; Xuereb et al., 2018). Despite this, geographic distance provides an adequate description of dispersal for many marine species (Berry et al., 2012; Hirase et al., 2020; Saenz‐Agudelo et al., 2012), and, in some species, distance is a better descriptor of dispersal than oceanography (Crandall et al., 2014; Wynsberge et al., 2017). Our study revealed both of these patterns in the same species: Distance provided a better explanation for dispersal than did ocean currents over part of our study domain, while ocean currents were necessary to explain dispersal elsewhere.

Part of our study region includes the Bohol Jet Current, a strong current that connects the western Pacific Ocean to the Sulu Sea and which flows past the southern tip of Leyte toward the southern tip of Cebu. Larvae dispersing south of Bohol in the southern part of our study region were likely influenced by this current, which would have induced much more rapid and directional dispersal than in the shallow strait between Bohol and Cebu or in the Camotes Sea north of Bohol. Our finding that potential connectivity strongly correlated with genetic distance between a site in southern Leyte and sites in southern Cebu suggests that the Bohol Jet Current is driving enhanced dispersal between these areas. This result corresponds with past findings of high connectivity from a biophysical model between southern Leyte and the south side of Bohol and Cebu and findings of similar species assemblages, suggesting the Bohol Jet Current is a major driver of westward larval dispersal, species diversity, and community composition in the Bohol Sea (Abesamis et al., 2016). Conversely, in our IBD study region in the shallow straits between Cebu and Bohol, ocean currents were weaker, relatively homogenous, stable (Figure S1), and likely difficult to model given the complex bathymetry. These factors may explain why geographic distance was the better explanation for patterns of genetic differentiation in this narrower region.

Regional ocean current patterns may also help explain why *A. biaculeatus* were found in low abundance or absent at sites off the northern coasts of Cebu and Leyte (see sites marked with Xs for low abundance and with triangles for zero abundance in Figure 1). The sites where *A. biaculeatus* were found at higher abundances fall into a connectivity cluster (an area in which ocean currents strongly connect a group of reefs) around the Bohol Sea, while the sites in which *A. biaculeatus* was rare or absent have been identified as part of a separate connectivity cluster that stretches from the northern Visayas north toward Luzon (Pata & Yñiguez, 2019; Thompson et al., 2018). The connectivity cluster identified around the Bohol Sea supports our findings of low pairwise *F*_ST_ among sites, as the sites are connected reasonably well with each other by currents. Due to these connectivity clusters, it is less likely that larvae from the high abundance sites would disperse to the northern sites. The northern sites are also well‐sheltered by the islands of Cebu and Leyte and current speeds are weaker than in the Bohol Sea (see Figure 1), which may reduce the chance of immigration from outside of our study area. These northern sites rank low in connectivity indices in an analysis of connectivity patterns in the Philippines (Pata & Yñiguez, 2021). The rarity of *A. biaculeatus* in the northern sites may also be partially explained by ecological factors, specifically competition for the *E. quadricolor* anemone. If *E. quadricolor* anemones are already occupied by other species of anemonefish (such as *A. clarkii* that was found at all sites in Figure 1), then *A. biaculeatus* larvae may not have suitable settlement habitat in that region.

For future dispersal studies, it will be important to determine the spatial extent at which geographic distance well explains larval dispersal among sites. Our results suggest that geographic distance may be most useful at narrow spatial extents, where ocean currents are more homogenous and oceanographic models are generally less accurate due to limitations on grid size and (in some parts of the world) a lack of fine‐scale bathymetric data. In contrast, ocean currents become a dominant explanation of dispersal at wider spatial extents. While some higher resolution models (grid sizes smaller than CT‐ROMS 5 × 5 km) do exist, they are very computationally intensive and thus are typically only be run over quite small spatial and temporal scales that are less useful for understanding regional larval dispersal. The CT‐ROMS model appears to be adequate for our study region, as its potential connectivity matrices were useful for explaining observed genetic structure.

Our finding of IBD patterns at small spatial extents and ocean current influenced dispersal at larger spatial extents corresponds with empirical examples of some long‐distance dispersal in addition to local IBD patterns in *Amphiprion omanensis* and in *Serranus cabrilla* (Benestan et al., 2021; Simpson et al., 2014). Future dispersal studies should take into account spatial extent when sampling and running analyses, as it may be prudent to run analyses in subsets according to spatial extent in addition to running all populations together. This approach will help elucidate the spatial extent at which IBD patterns are relevant for each species and geographic region. As we did find a general trend between potential connectivity and genetic distance at wider spatial extents, potential connectivity analyses could be run in other regions to further understand dispersal of *A. biaculeatus* and other coral reef species, as it is likely other species will be similarly influenced by currents (Benestan et al., 2021; Bode et al., 2019). Additionally, it is important to consider the heterogeneity of ocean currents in a study region, as areas with homogenous ocean currents will likely follow IBD patterns, while areas with spatially heterogeneous currents may require oceanographic analysis.

### 4.4 Conservation and management implications

Our dispersal spread estimate for *A. biaculeatus* can be useful for designing marine reserve networks that benefit coral reef species and fisheries. Anemonefish are useful proxies to use in reserve design as they are widespread throughout the Indo‐Pacific region and have a dispersal spread that is similar to or longer than many coral reef fishes, meaning most fishes will see spillover effects from reserves designed around the 9 km dispersal spread we found for *A. biaculeatus*. Reserves have been shown to contribute substantially to juvenile recruitment both inside and outside of reserves in commonly fished coral reef species such as trout and snapper (Harrison et al., 2012). To successfully protect and increase a single population, theory suggests that isolated marine reserves should be roughly twice as wide as a species' dispersal spread (i.e., 18 km for *A. biaculeatus* in the Philippines) or, alternatively, be part of a network of smaller marine reserves spaced closely together (Gaines et al., 2010; Harrison et al., 2012; Lockwood et al., 2002). If maximum population growth inside and outside of reserves is the goal, guidelines suggest spacing reserves at or below the mean dispersal distance (Gaines et al., 2010). If the primary goal is to have a spillover effect outside the reserve, then small reserves could be spaced closely together. Southeastern Cebu and most of Bohol have a network of closely spaced reserves (<15 km apart) that should support ample spillover of *A. biaculeatus* and most coral reef fish species (Cabral et al., 2014). Many of these reserves have an area of less than 1 km^2^, which may protect anemonefish and species whose adult stage is nonmobile but may not be adequate for species with higher adult mobility (Weeks et al., 2010). The distribution of reserves is not uniform across the Philippines and the Coral Triangle region, and most reserves are spaced too far apart to benefit species with short and intermediate dispersal distances (Abesamis et al., 2017; Gaines et al., 2010). Our findings support a broader effort to establish closely spaced marine reserve networks across the Indo‐Pacific. Our recommendations for *A. biaculeatus* are similar to recommendations from a review by Green et al. (Green et al., 2015) that concluded coral reef reserves should be less than 15 km apart. Designing closely spaced reserve networks may also have long‐term benefits to reefs in the face of climate change because dispersal spread is predicted to decrease as ocean temperatures rise (Álvarez‐Romero et al., 2018; O'Connor et al., 2007).

Regional connectivity patterns can also inform reserve design. The general agreement we found between potential connectivity derived from oceanographic models and genetic distance at wider spatial extents suggests that CT‐ROMS potential connectivity matrices can be applied toward designing marine reserves in other regions and species of the Coral Triangle. Researchers and managers could collaborate and use the matrices to understand current flows in their region of interest and design reserves accordingly. In regions with high connectivity, having multiple small, closely spaced reserves may be sufficient, while more isolated areas may benefit from a larger reserve that is self‐sustaining (Abesamis et al., 2016). Additional studies would also be useful to further test the utility of oceanographic currents for explaining larval dispersal across a wider range of species and oceanographic regions.

### 4.5 Conclusions and future directions

Our study approach and methods can be applied across marine taxa to estimate dispersal spread and to better understand the drivers behind marine larval dispersal across spatial extents. We recommend future studies consider multiple spatial extents to gain a better understanding of the spatial extent of IBD in a wider diversity of species and environments. Future studies evaluating the congruence between indirect IBD and direct parentage estimates of dispersal spread would also be useful to understand the utility of indirect methods across a wider range of marine taxa. Using Rousset's equation (Rousset, 1997) for calculating dispersal spread using *N*_e_ and the slope of the linear regression between linear *F*_ST_ and geographic distance is restricted to species that follow IBD patterns, but as this covers more than half of marine taxa studied to date (Selkoe et al., 2016; Wright et al., 2015), it can be a useful method in many study systems. We used Rousset's equation for one‐dimensional habitats, which is suitable for many coastal species, whereas equations for two‐dimensional habitats are likely more useful for atoll or pelagic landscapes. Our study approach can be easily adapted to utilize newer sequencing technologies such as ddRAD or whole‐genome sequencing (Benestan et al., 2021; Saenz‐Agudelo et al., 2015). Estimating *N*_e_ remains one of the largest remaining sources of uncertainty in IBD calculations, and additional work refining linkage disequilibrium and other methods would improve our ability to make precise dispersal estimates.

We also recommend evaluating the correlation between genetic distance and a measure of the physical drivers of dispersal, such as potential connectivity, when possible to better understand the primary influence on dispersal for different species. Ocean currents may be less useful to explain dispersal for species with different larval behaviors, for example. The potential connectivity analysis we used can be applied to any species and area that has oceanographic modeling data. Potential connectivity matrices are available for the entire Coral Triangle region (Thompson et al., 2018), making this feasible for researchers and managers in the region. Using IBD and isolation‐by‐oceanography analyses in tandem and across multiple spatial extents can provide vital dispersal information and inform the development and implementation of more successful conservation actions.

## CONFLICT OF INTEREST

The authors declare no conflicts of interest.

## Supporting information


Figure S1

Table S1

Appendix S1

## Data Availability

The data and code that support the findings of this study are openly available at https://doi.org/10.5281/zenodo.6908607.
