# Supplementary figures and images for: Isolation‐by‐distance and isolation‐by‐oceanography in Maroon Anemonefish (Amphiprion biaculeatus)
Source: Evol Appl. 2022 Aug 25;16(2):379–92. doi: 10.1111/eva.13448 (PMC9923474; doi:10.1111/eva.13448)

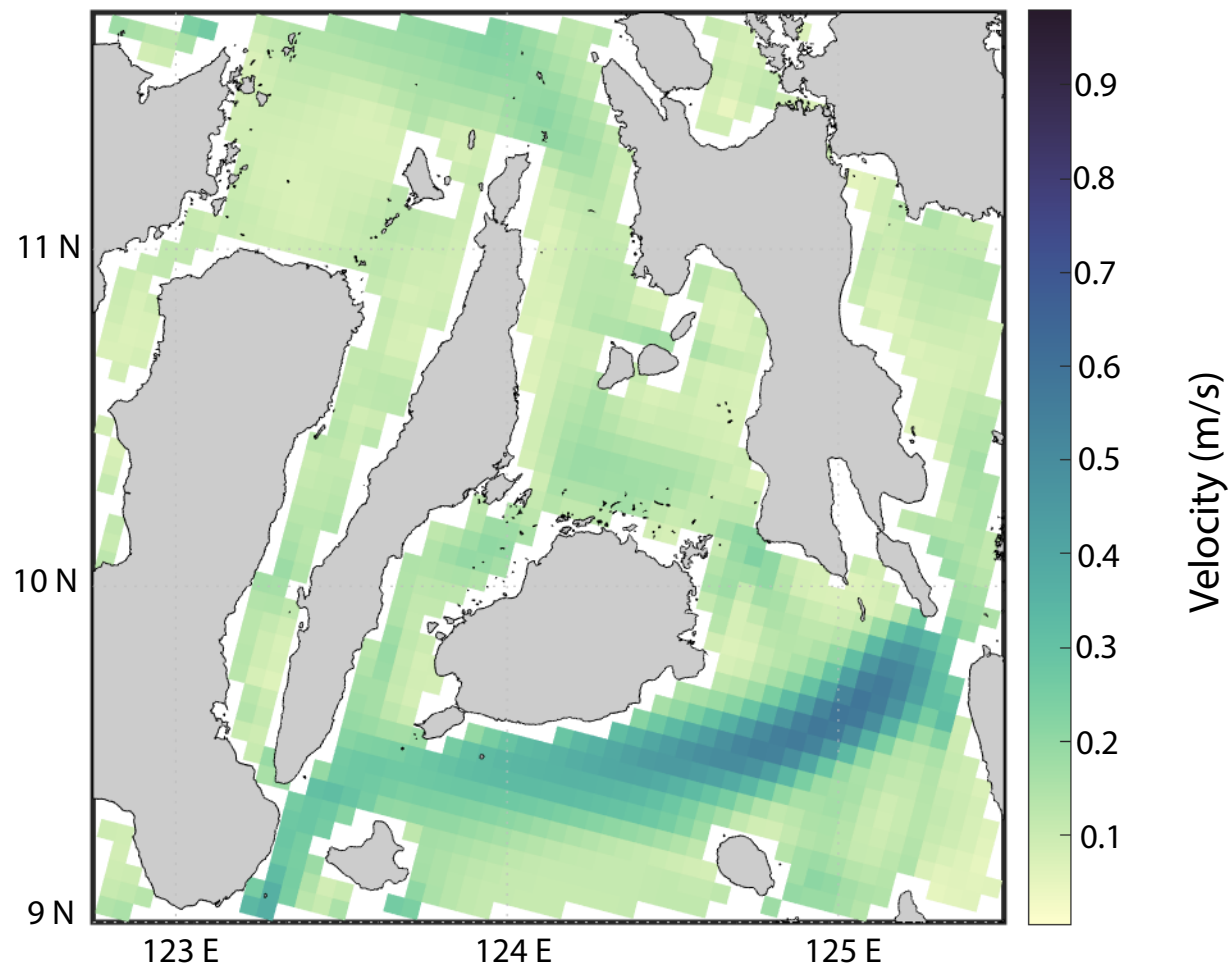

Supplement: Supplementary file 1 — Figure S1 [file EVA-16-379-s003.pdf]
