# Supplementary material for: Isolation‐by‐distance and isolation‐by‐oceanography in Maroon Anemonefish (Amphiprion biaculeatus)
Source: Evol Appl. 2022 Aug 25;16(2):379–92. doi: 10.1111/eva.13448 (PMC9923474; doi:10.1111/eva.13448)
Supplement: Supplementary file 2 — Table S1 [file EVA-16-379-s002.docx]

**Supplementary Material**

**Table S1.** Forward and reverse primer sequences for unpublished loci obtained via personal communication from G. Bernardi.

| **Locus** | **Forward Primer Sequence** | **Reverse Primer Sequence** |
| --- | --- | --- |
| ACH_A11 | TGC-TCG-TCT-ACT-GGT-TTC-G | TTC-ACC-CCT-TCA-CTC-AGC |
| ACH_A4 | TTG-TTA-CTG-TGT-CCG-TGT-GAT-C | GGC-GAC-ATG-ATA-CAC-TTG-ACT-T |
| ACH_B9 | ACT-TCC-AAC-GCA-ATG-ACT-TC | AAA-GCA-CAC-CCA-AGA-GAA-TG |
| ACH_A7 | AGG-GAA-AAG-TAA-TAC-AAC-GAG-C | CAG-CAC-AGG-AAA-ACA-GAG-G |
| ACH_C1 | GCG-ACC-TTG-TTA-TCA-CTG-TC | TTG-GTT-GGA-CTT-TCT-TTG-TC |
| ACH_D1 | CCA-AAA-GTT-TAG-GAA-GCT-ACC | AAC-CAG-ACT-GCC-CTG-ATA-C |
| ACH_A3 | GGT-TGC-AGT-TTT-TAA-CAG-GAC | GCC-CTA-CAT-CAG-GTA-CAC-AAC |
| ACH_A8 | ACA-AGG-GTC-TGA-GAG-AGC-TAC | TGG-TCA-CTG-TGA-GGT-AAT-CAG |
