# Supplementary material for: Isolation‐by‐distance and isolation‐by‐oceanography in Maroon Anemonefish (Amphiprion biaculeatus)
Source: Evol Appl. 2022 Aug 25;16(2):379–92. doi: 10.1111/eva.13448 (PMC9923474; doi:10.1111/eva.13448)
Supplement: Supplementary file 3 — Appendix S1 [file EVA-16-379-s001.docx]

**Figure Legend**

Figure S1 Plot of standard deviation of velocity from January 2003 to December 2007 in m/s in the central Philippines.
